# Statins and immunotherapy: Togetherness makes strength The potential effect of statins on immunotherapy for NSCLC

**DOI:** 10.1002/cnr2.1368

**Published:** 2021-03-31

**Authors:** Alessandro Rossi, Marco Filetti, Beatrice Taurelli Salimbeni, Marta Piras, Francesco Rizzo, Raffaele Giusti, Paolo Marchetti

**Affiliations:** ^1^ Department of Clinical and Molecular Medicine, Oncology Unit “La Sapienza” University of Rome, Azienda Ospedaliera Sant'Andrea Rome Italy

**Keywords:** immunotherapy, lung cancer, NSCLC, statins, survival

## Abstract

**Background:**

Recent researches suggested that statins, beside their role in inhibiting endogenous cholesterol synthesis and in cardiovascular prevention, could influence several processes in cancer biology. In fact, a recent meta‐analysis demonstrated that statins could positively influence OS in lung cancer patients.

**Aim:**

There is a lack of large cohort studies that could support a potential antineoplastic role of statins in clinical practice. We collected data from 162 patients treated with immunotherapy for Nonsmall Cell Lung Cancer (NSCLC) in first‐ and second‐line setting to investigate the impact of these drugs on survival parameters.

**Methods and Results:**

In our observational study, we enrolled 162 patients who received immunotherapy for lung cancer between October 2015 and April 2020. We used descriptive statistics to analyze patients' baseline features. Tumor response was evaluated using RECIST version 1.1 guidelines. Uni and multivariate analysis were conducted to investigate the relationship between statin use and response to immunotherapy, using the χ^2^‐test. We used Kaplan‐Meier curves to estimate OS and PFS in statin and nonstatin users. We included 122 patients in the final analysis. Median PFS was 17.57 months in the statin group and 9.57 months in the nonstatin group, with a *P* = <.001. Moreover, median OS was superior in the statin‐users group, with a statistically significant difference (19.94 vs 10.94 months, *P* = <.001).

**Conclusion:**

Although in our study, statin use positively correlates with PFS and OS in lung cancer patient treated with immunotherapy, these results require a further validation with randomized clinical trials.

## 1 INTRODUCTION

Statins are commonly used agents in the primary and secondary prevention of cardiovascular disease.^1^ Recent studies suggested that they could display pleiotropic effects on several cancer‐related cellular processes, such as proliferation, apoptosis, angiogenesis, and metastasis.^2^ Even though the promising molecular features, results of randomized clinical trials investigating the combinations between statins and anticancer treatments have been controversial so far,^3^, ^4^ especially on lung cancer, the most common and deadly neoplasm worldwide.^5^ Despite the impressive advances in the management of this disease, the 5‐year survival rate is still at 18%.^5^, ^6^ In metastatic nonsmall cell lung cancer (NSCLC) immunotherapy, along with targeted therapy, increased progression‐free (PFS) and overall survival (OS).^6^, ^7^, ^8^, ^9^, ^10^, ^11^, ^12^ A recent meta‐analysis suggested that statins could positively affect the risk of all‐cause mortality and improve OS in lung cancer patients^13^; conversely, no influence on PFS and overall response rate (ORR) was observed. Statins exhibit an immunomodulatory effect by preventing protein prenylation,^14^ and this leads to increased antigen presentation, T‐cell activation, and cytolytic response. Prenylation creates a hydrophobic region that determines protein attachment to the membrane and enables their optimal functioning. Proteins of key signaling pathways that are overactivated in many types of cancer, such as those from Ras, Rho, and Rab superfamily, are prenylated; therefore, preventing the prenylation branch could be a potential strategy in cancer treatment.^15^, ^16^ This suggested that these drugs could synergize with immunotherapy in the treatment of lung cancer.^17^

The introduction of immune checkpoint inhibitors (ICIs) has revolutionized the treatment of advanced NSCLC. Almost all patients are treated in the first‐ or second‐line setting with immunotherapeutic agents, alone or in combination with other cytotoxic drugs. Not all patients respond in the same way to immunotherapy, and peculiar response patterns could be observed in some cases, such as pseudo or hyperprogression.^18^ Alongside hyperprogression, a rare phenomenon that consists in primary resistance to treatment with a paradoxical and abnormal increase in tumor growth, secondary resistance to therapy after an initial excellent response, is more frequently observed. The reasons why ICIs lose their effectiveness after a variable length of time are not fully understood. Mechanisms that alter homeostasis between the tumor and the immune system probably come into play, with lymphocyte depletion and consequent tumor escape. Analyzing the possible impact of preexisting therapies on the activity of ICIs and the homeostasis of tumor microenvironment becomes of crucial importance. To our knowledge, only one study conducted on patients with malignant pleural mesothelioma and NSCLC investigated the possible role of statin statins in patients treated with ICI.^19^ Given the encouraging in vitro and in vivo results,^13^, ^14^, ^17^ a retrospective analysis was performed on 162 patients affected by metastatic NSCLC treated with ICIs to determine the impact of statin on survival outcomes.

## 2 METHODS

In this observational retrospective study, we enrolled 162 patients affected by metastatic NSCLC treated at our institution between October 2015 and April 2020. Patients were eligible if complete data on clinical features, treatment, and survival outcomes were available. Inclusion criteria were:

- Histologically confirmed diagnosis of stage IV NSCLC without oncogenic driver mutations (eg, EGFR, ALK, ROS1)
- Immune‐checkpoint inhibitor (ICI) treatment in I or II line (patient in second line must have received a platinum‐based chemotherapy)
- Age ≥ 18 years
- ECOG performance status ≤2;
- Adequate renal, hepatic, and bone marrow function
- At least four administrations of immunotherapy‐containing regimen.

Patients without an available radiological tumor response evaluation using the immune Response Evaluation Criteria in Solid Tumors (iRECIST)^20^ were excluded. All patients were candidates for ICI treatment (allowed protocols: Pembrolizumab 200 mg IV infusion in 100 mL NS every 3 weeks; Nivolumab 240 mg IV in 100 mL NS every 2 weeks; Atezolizumab 1200 mg IV in 100 mL NS every 3 weeks), according to the national guidelines. Demographic data, medical history, and adverse drug reactions were collected. We used medical records to assess statin use, defined as the use of these drugs for at least 1 month before starting treatment. PD‐L1 tumor status was determined using the VENTANA PD‐L1 SP‐142 clone (Ventana Medical Systems Inc., Tucson, AZ) and the PD‐L1 IHC 223 pharmDx DAKO OMNIS (Agilent Technologies, Inc., Santa Clara, CA). We also recorded delays and permanent discontinuations. Primary endpoints were OS and PFS, while secondary endpoints were ORR and immune‐related adverse events (IRAEs) across statin and nonstatin groups. PFS was defined as the time from treatment start to that of disease progression or death. OS was defined as the time from treatment start to death from any cause or last follow‐up. ORR was defined as the proportion of patients who have a partial or complete response (CR) to therapy. IRAEs were defined as inflammatory side effects due to an exuberant activation of the immune system, and graded with Common Terminology Criteria for Adverse Events (CTCAE) v5.0.^21^

The study was conducted following the Declaration of Helsinki. Due to the retrospective nature of the study, Institutional Review Board approval was obtained before the divulgation of scientific data.

### 2.1 Statistical analysis

Descriptive statistics were used to analyze patients' baseline features. Categorical variables were addressed by χ^2^ or Fisher exact test. The Kaplan‐Meier method and log‐rank test were used to estimate survival and compare the inherent data across the defined subgroups. We used univariate and multivariate Cox proportional hazards regression models to evaluate associations of clinic pathologic features with PFS and OS. SPSS software (SPSS version 21.0, SPSS Inc., Chicago, IL) was used for all statistical evaluations. The significance levels for all performed tests was set at *P* < .05.

## 3 RESULTS

**FIGURE 1:**
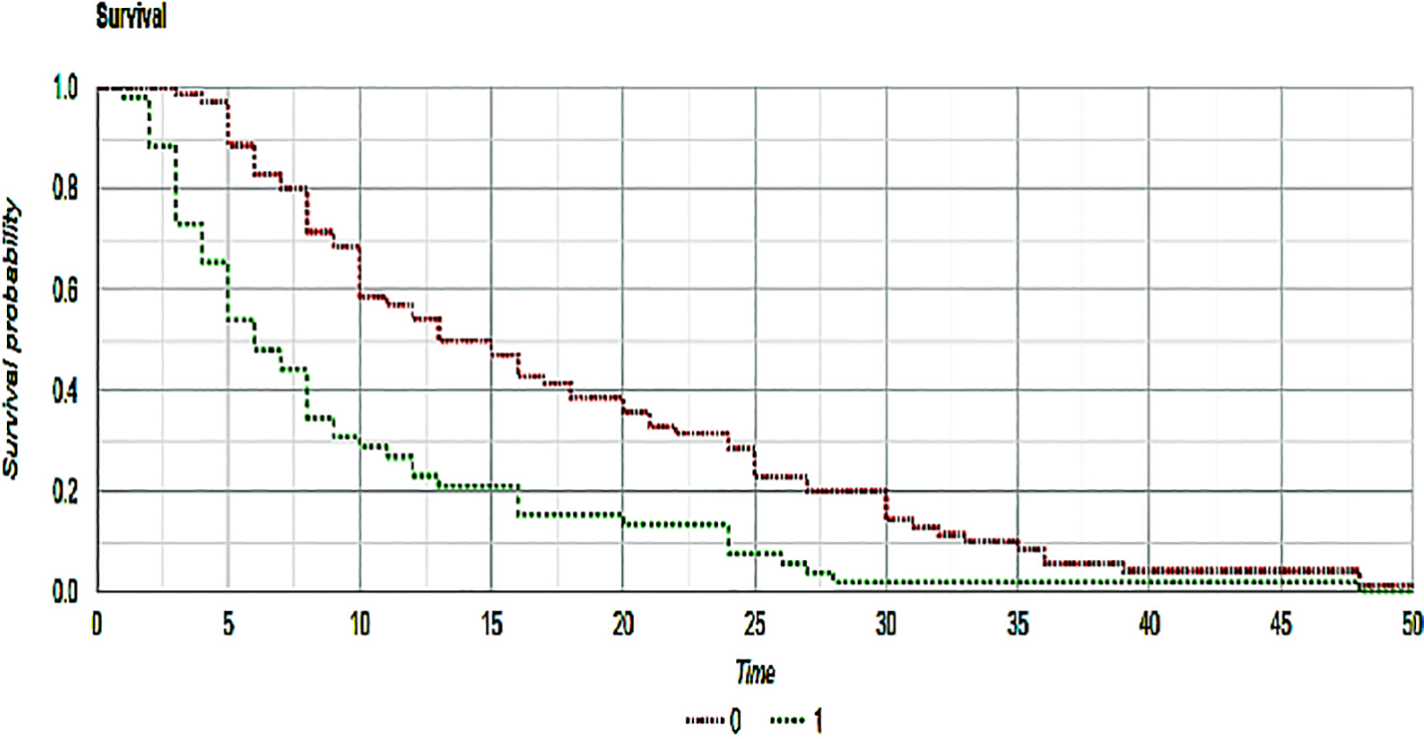
Kaplan–Meier curves that show overall survival of statin (red) and nonstatin (green) patients. *P*‐value between the two groups was <.001

**FIGURE 2:**
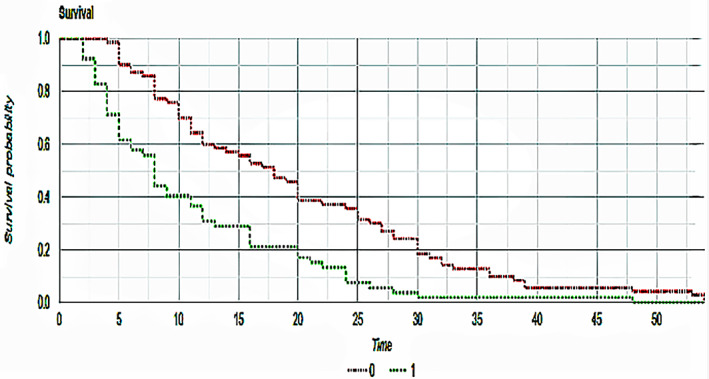
Kaplan–Meier curves that show progression‐free survival of statin (red) and nonstatin (green) patients. *P*‐value between the two groups was <.001

Among all 162 screened patients, 122 met the requested criteria (52 in the non‐statin group, 70 in the statin group) and were included in the final analysis. Clinical features of the global study population are summarized in Table 1. According to the iRECIST criteria, we observed 5 CRs (3 in the non‐statin group, 2 in the statin group), 42 partial responses (10 in the non‐statin group, 32 in the statin group), 53 stable diseases (SDs) (22 in the non‐statin group, 31 in the statin group) and 22 progressive diseases (PDs) (17 in the non‐statin group, 5 in the statin group). Rates of partial responses (PR) were statistically significant between statin and non‐statin users (19.23% in the non‐statin group, 45.71% in the statin group, *P* = <.001); also rates of PDs were in favor of statin users, with a meaningful difference across the two groups (32.69% vs 7.14%, respectively, *P* = <.001). There were no significant differences between CR and SD rates over the two groups (*P* = .317 and *P* = .78, respectively). Median OS was superior in the statin‐users group, with a statistically significant difference (19.94 vs 10.94 months, *P* = <.001) as shown in Figure 1. Median PFS was 17.57 months in the statin group and 9.57 months in the non‐statin group (*P* = <.001) as shown in Figure 2. ORR was 48.56% in the statin group, and 24.99% in the non‐statin group, with a statistical significance (*P* = <.001). Among the statin group, 26 patients had any grade toxicity (37.14%), with five patients experiencing a G3‐G4 toxicity (7.14%). Four of these (5.71%) required permanent treatment discontinuation. Among the non‐statin group, 18 patients had any grade toxicity (34.61%), with four patients experiencing a G3‐G4 toxicity (7.84%). Two of these (3.92%) required permanent treatment discontinuation. Notably, neither any grade nor the G3‐G4 toxicity rates differed across the two groups (*P* = .709 and *P* = .851, respectively). Details about reported toxicities are listed in Table 2.

**TABLE 1:** Patients' baseline features

Parameters	Statin users (n=)	Nonstatin users (n=)
Age, years [median (range)]	71 (48‐93)	70 (46‐90)
Sex		
Male	60	46
Female	28	28
Smoking habit		
Current‐former	72	66
Never	16	8
Performance status		
0–1	80	62
2	8	12
Histology		
NSqNSCLC	47	45
SqNSCLC	39	22
Other histology	2	7
PDL1 (%)		
0	11	17
1%–49%	5	8
≥50%	44	26
Not available/Not tested	28	23
IT line of treatment n.		
1	40	24
2	48	50
Drugs		
*Pembrolizumab*	45	30
*Nivolumab*	34	30
*Atezolizumab*	9	14

Abbreviations: IT, Immunotherapy; NSCLC, Nonsmall cell lung cancer; NSq, Nonsquamous; PDL1, Programmed death‐ligand 1; Sq, Squamous.

**TABLE 2:** Reported toxicities

	Number of patients (n=)	Any grade toxicity (n=)	Type of toxicity	G3‐G4 toxicity (n=)	Type of toxicity	Treatment discontinuations (n=)
Statin users	70	26	Hypothyroidism (7) Hyperthyroidism (6) Dermatitis (4) Diarrhea (2) Hepatitis (1) Arthritis (2) Pneumonitis (3) Allergic reaction (1)	5	Pneumonitis (2) Diarrhea (1) Arthritis (1) Allergic reaction (1)	4
Nonstatin users	52	18	Hypothyroidism (4) Hyperthyroidism (1) Dermatitis (1) Psoriasis (1) Diarrhea (1) Arthritis (6) Pneumonitis (1) Dyspnea with bronchospasm (1) Vitiligo (1) Asthenia (1)	4	Hypothyroidism (1) Dyspnea with bronchospasm (1) Arthritis (1) Psoriasis (1)	2

## 4 DISCUSSION

We sought to examine the possible role of statins in patients treated with ICI in first and second‐line setting. Our results seem to be in contrast with most of the available body of evidences, where no statistical significance in PFS and ORR was found between statin and non‐statin patients. Data regarding OS are instead in line with what was traditionally reported, with a positive effect of statins in cohort and case‐control studies, but not in randomized clinical trials.

In particular, Cantini et al^19^ demonstrated an advantage in PFS and ORR parameters when statins were used along anti‐PD1 agents for NSCLC in second‐line setting, after progression on standard chemotherapy. Conversely, no influence on OS was recorded. It is important to note that only 36 out of 130 (20%) received statins, so this could have led to possible bias. Omori et al^17^ also demonstrated a positive survival trend in patients treated with Nivolumab and statins in second‐line setting, but only 10% of the entire cohort was receiving the lipid‐lowering therapy, so this observational study may have suffered from low data power.

Using data from the Surveillance, Epidemiology and End Results registry (SEER), Lin et al^22^ conducted an interesting analysis on 5118 patients >65 years of age diagnosed with metastatic NSCLC and treated with chemotherapy, revealing that there was a statistically significant advantage in OS for patients receiving statins (27% of the entire cohort, 1404 patients). Although our study focused its target on the combination between immunotherapy and statin use, this large cohort may help understanding the real impact on survival of these drugs. Results should be interpreted with caution, because author did not evaluate the effect on younger patients, an eventual dose‐dependent effect or the adherence to therapy.

In our study, 48 patients received first‐line Pembrolizumab as a single agent therapy due to the strong expression of PD‐L1 (≥50%) and, among these, 34 (70.84%) were assuming statins, while 14 (29.16%) were not. Notably, rates of PFS and OS did not differ significantly in this subgroup of patients (median PFS 14.44 months in statin group vs 15.5 months in nonstatin group, *P* = .8340, and median OS 16.23 months vs 15.85 months, respectively, *P* = .9418). Seventy‐four patients with moderate (1%‐49%) or absent (0% or not determined) PD‐L1 expression received a second‐line ICI after progression under standard platinum‐based chemotherapy, with 36 of these taking statins. Both PFS and OS resulted as statistically relevant when the two groups were compared (median PFS 20.66 months in statin group vs 7.39 months in nonstatin group, *P* = .007, and median OS 23.44 months vs 9.13 months, respectively, *P* = .0063). This confirmed an advantage on survival parameters in the second‐line setting with patients previously exposed to chemotherapy. Although second‐line Pembrolizumab and Nivolumab subgroups were balanced toward the number of patients enrolled (5 vs 5 and 18 vs 21, respectively), the Atezolizumab subgroup presented only three patients assuming statins and 12 patients without the lipid‐lowering therapy. Notably, this particular subgroup had the worst PFS (9.66 months in statin group vs 5.08 in nonstatin group, *P* = .21) and OS (9.66 months vs 7.4 months, respectively, *P* = .56, data not shown). This may have led to a survival outcome's decrease in the nonstatin group, and should be taken into account. Taken together, these results suggest that second‐line, rather than first‐line, ICI treatment combined with statins could positively affect survival outcomes in patients affected by metastatic NSCLC. Our study certainly presents some limitations. First, due to its observational nature, it is more prone to selection biases, and results should be interpreted with caution. Second, statin‐based therapy was investigated before diagnosis, and we did not assess the impact of an eventual post‐diagnosis use on survival outcomes. Third, although the well‐known differences in pharmacokinetic profiles among statins, we did not discriminate between hydrophobic and hydrophilic compounds.

## 5 CONCLUSION

Immunotherapy, alone or in combination with standard chemotherapy regimens, has dramatically changed the landscape of lung cancer treatment over the last years.^9^, ^23^, ^24^ Despite promising results, we need to better understand the complex interaction between the immune system and cancer microenvironment to achieve better outcomes and durable responses.^25^ In our study, we demonstrated a significant relationship between improved PFS and OS and pre‐existing statin use. Although interesting, this result needs to be validated with randomized clinical trials and larger cohorts (to select which type of patients could benefit the most with this pharmacological association). To date, many efforts have been made to ameliorate lung cancer patients prognosis and quality of life, even with the use of nonconventional anticancer drugs beside available therapies^26^, ^27^; our study provides a useful hint in this intricate scenario and paves the way for a new use of an already worldwide available treatment.

## CONFLICT OF INTEREST

The authors declare no conflict of interest.

## ETHICAL STATEMENT

Institutional Ethical Review Board approval was obtained before the divulgation of scientific data, with ID 5585_2019. Ethical Body responsible for approval: Ufficio locale Sperimentazioni Cliniche, Comitato Etico dell'Università Sapienza (RM). Azienda Ospedaliero‐Universitaria Sant'Andrea, Via di Grottarossa, 1035‐1039 ‐ 00189 Roma.

## AUTHOR'S CONTRIBUTIONS

All authors had full access to the data in the study and take responsibility for the integrity of the data and the accuracy of the data analysis. *Conceptualization*, A.R., M.F., R.G.; *Methodology*, A.R., M.F., R.G.; *Investigation*, A.R., M.F., R.G.; *Formal Analysis*, A.R., M.F.; *Resources*, A.R., M.F., M.P., F.R., R.G.; *Writing ‐ Original Draft*, A.R., M.F., R.G.; *Writing ‐ Review & Editing*, A.R., M.F., B.T.S., M.P., R.G.; *Visualization*, A.R., M.F.; *Supervision*, A.R., M.F., B.T.S., R.G., P.M.; *Data Curation*, A.R., M.F., B.T.S., M.P., F.R.; *Project Administration*, A.R., P.M.; *Software*, A.R., M.F.; *Validation*, A.R., M.F.

## Data Availability

The data that support the findings of this study are available on request from the corresponding author. The data are not publicly available due to privacy or ethical restrictions.
